# Mindsets and self-efficacy beliefs among individuals with type 2 diabetes

**DOI:** 10.1038/s41598-023-47617-4

**Published:** 2023-11-21

**Authors:** Carolyn J. Lo, Leonard Lee, Weichang Yu, E Shyong Tai, Tong Wei Yew, Isabel L. Ding

**Affiliations:** 1 Yong Loo Lin School of Medicine, National University of Singapore, Singapore, Singapore; 2 LRF Institute for the Public Understanding of Risk, National University of Singapore, Singapore, Singapore; 3 Department of Marketing, NUS Business School, National University of Singapore, Singapore, Singapore; 4 School of Mathematics and Statistics, University of Melbourne, Melbourne, Australia; 5 Department of Medicine, National University Hospital, Singapore, Singapore; 6 Warwick Business School, University of Warwick, Coventry, UK

**Keywords:** Psychology, Endocrinology, Endocrine system and metabolic diseases, Diabetes

## Abstract

Growth mindsets and self-efficacy beliefs have been known to predict and promote resilience, challenge seeking, and improved outcomes in areas such as education and intelligence. However, little is known about the role of these two potentially influential beliefs in the context of type 2 diabetes (T2D), specifically in terms of whether and in which domains (i.e., beliefs toward general life, general health, or condition-specific domains) these beliefs—or lack thereof—is prevalent among individuals with T2D. Given the lifelong challenges that individuals with diabetes often encounter with managing their disease, many may slip into a conceding negative belief that their diabetes is “too difficult to control” or simply “out of their hands,” inhibiting proactive self-management efforts. Results from our study (*n* = 893) revealed that individuals with T2D had a significantly lower growth mindset towards their blood glucose level and lower self-efficacy towards their general health, blood glucose, and cholesterol levels compared to those without T2D. Among participants with T2D, further analyses showed a pattern of higher HbA1c among those with lower growth mindsets and self-efficacy toward their general health or blood glucose level. These findings identify the belief-domains that may pose barriers to necessary self-care behaviors, informing future interventions to promote improved diabetes care and management.

## Introduction

The prevalence of type 2 diabetes (T2D) has become a serious threat to global health, with many diagnosed individuals experiencing significant challenges in maintaining adequate glycemic control^1^. Although evidence-based lifestyle changes and pharmacotherapy can help reduce mortality and the risk of diabetes-associated complications, the success of such interventions hinges largely on individuals’ incorporating self-management behaviors in their lives. Many of these behaviors, however, are difficult to sustain over the long-term and require individuals to navigate complex barriers throughout their lifetime^2^. Those who initially endeavor to modify their lifestyles may often revert to previous habits, especially after encountering setbacks such as increased inconvenience, conflict with personal priorities, and unsatisfactory blood glucose control. Should unfavorable health outcomes persist over time, individuals may slip into a conceding belief that their diabetes is “too difficult to control,” that they must be “genetically prone” so there is little they can do to change, or that their diabetes is simply “out of their hands.” Clearly, setbacks in self-management comprise only part of the various factors that may lead to failure in blood glucose control. But it appears that beliefs that one’s diabetes—or more specifically, blood glucose—cannot be easily improved, controlled, or managed may be quite pervasive, offering a plausible perspective of individuals’ difficulties in achieving sustained glycemic control through self-management. Obtaining an empirical understanding of this conjecture is pivotal in laying the groundwork for novel strategies to support behavioral change as has been demonstrated in other domains like education^3^.

## Health beliefs: Mindsets and self-efficacy

Research in the fields of psychology and healthcare suggests that people’s health-related behaviors are often influenced by their beliefs and expectations^4–6^. In particular, fixed versus growth mindsets and self-efficacy (SE) beliefs hold especial relevance in contexts fraught with challenges^7,8^. Individuals with a growth mindset (GM) believe that a specific attribute or condition is malleable, leading them to embrace adaptive beliefs and behaviors (e.g., believing that improvements stem from effort rather than existential abilities or genes, viewing failures as opportunities for growth, learning from setbacks, and responding constructively to difficulties)^9^. At the other end of the spectrum, individuals may have a fixed mindset (FM), believing that there is little they can do to change or improve within a certain area of function, which is further reinforced by poor performance or outcomes^10^. Individuals may hold differing mindsets in different domains (e.g., a GM toward athletics but a FM toward math), with mindset valence varying across the fixed-growth spectrum^11,12^.

Although mindsets have been well researched in the domains of education and intelligence (e.g., GM has been observed to improve academic achievement^13,14^) and, increasingly, in health-related domains (e.g., weight management^15^, smoking addictions^16^, healthier eating intentions^17^), there has been limited research pertaining to diabetes. To our best knowledge, only one correlational study involving 91 adolescents with type 1 diabetes has reported the role of GM in the context of diabetes: compared to adolescents with a FM, those with a GM had better glycemic outcomes after transitioning to adult care, with greatest improvements in HbA1c (the blood test used to assess glycemic control) among GM-adolescents who had the poorest glycemic control (HbA1c > 10%) before transition^18^. Taken together, extant theoretical and empirical research indicating that a GM can predict and promote more resilience, challenge seeking, and positive outcomes^19^ suggests that instilling a GM among individuals with T2D pertaining to the control of their disease may prove a crucial precursor to successful diabetes management. Thus, there is much potential for evidence-based research to establish whether and where mindsets matter in the context of T2D.

In contrast, SE has received more attention in the healthcare literature, including diabetes. Referred to as one’s beliefs in their capabilities to execute behaviors required to achieve a desired outcome, SE influences how much effort and persistence individuals will exhibit in the face of obstacles^8^. SE has been found to be positively associated with health outcomes in chronic illnesses^20,21^ and is widely accepted as a determinant of motivation and self-care behaviors in individuals with diabetes and obesity^22^. In a systematic review and meta-analysis of 739 correlational studies on T2D, SE was consistently associated with self-care behaviors including adherence to diet, physical activity, medications, and appointment keeping. Given that individuals with T2D regularly encounter new, demanding and/or difficult situations when managing their diabetes, the belief that they have the capacity to handle such challenges is important for effective self-management.

Although some research has found GM and SE to be positively correlated^23,24^, they are conceptually distinct. GM are beliefs or “frames of mind” that a specific attribute/condition can be *developed* or *changed* with effort and over time^19^. SE, on the other hand, does not focus on a condition’s malleability but instead pertains to individuals’ beliefs about their *ability* to perform specific behaviors. This sense of ability can be acquired through mastery experience, vicarious experience, verbal persuasion, or physiological feedback^8^. While it might be expected that someone with high GM would also likely have high SE, these two constructs should not be conflated. Individuals may have high GM but low SE where they believe that their medical condition is malleable while feeling that they lack the ability to successfully manage their condition.

At this juncture, we recognize that GM and SE are not the only belief constructs pertinent to diabetes management and glycemic control. Health-related psychological constructs such as health locus of control (LOC) and outcome expectancies may seem to share some similarities with GM as their positive orientations may predict, or at least be associated, with health outcomes. Nonetheless, these constructs have clear conceptual distinctions. Health LOC refers to beliefs about whether one’s health outcomes are attributed to internal factors (personal control) or to external factors (powerful others, fate, or luck)^25^. In contrast, GM is less concerned with assigning responsibility for outcomes and instead centers on the belief of the malleability of a condition or attribute and the willingness to undertake challenges as a result of that potential for improvement. Outcome expectancies primarily focus on beliefs that a given behavior will lead to certain expected (desired) results^8^, while GM emphasizes the process of effort and willingness to learn through setbacks rather than necessarily arriving at a desired outcome. Compared to GM, health LOC and outcome expectancies have been relatively well studied in cohorts with diabetes and may have limited potential for generating novel contributions in our current context. Notably, research on these constructs has reported mixed findings. A meta-analysis of health LOC, for instance, found weak correlation between external LOC and HbA1c and no correlation between internal LOC and HbA1c^26^. This led the authors to recommend excluding the use of health LOC measures in the design of diabetes care protocols. In another study, outcome expectancies were found to be only moderately correlated with diabetes self-care compared to self-efficacy^27^, while yet another study found that dietary outcome expectancies failed to predict dietary self-care^28^.

Given the presently limited research on GM and the prominence of SE as a recognized mechanism of diabetes outcomes^28^, there is substantial opportunity for examining beliefs that may hold greater resonance and applicability in the context of diabetes management. Importantly, investigating both GM and SE allows us to explore the role of two potentially influential beliefs in the T2D context in order to better ascertain the patient segment needing additional support, and where and how that support may be directed. Thus, we aim to (1) examine whether individuals with T2D would tend to have less GM and lower SE compared to individuals without T2D and (2) identify the domains in which differences in beliefs are observed. Assessing psychological constructs at more general, broader-level domains may be suited for predicting more general patterns of behavior or outcomes across multiple contexts^29^. On the other hand, measuring beliefs at a more specific level may be more optimal for predicting specific behaviors and outcomes within a specific context^8^. Given the advantages of both broader and specific measures, we examined beliefs in *general life* and *general health* domains, as well as in condition-specific areas—namely *blood glucose*, *blood pressure*, and *cholesterol* levels (three key clinical parameters that need to be optimized to manage diabetes effectively^1,30^). Of these, blood glucose is often more difficult to successfully control given that medication adherence alone is typically insufficient and must be accompanied by sustained lifestyle modifications^31,32^.

We also examine how GM and SE beliefs toward one’s blood glucose and general health may correspond with blood glucose (measured using HbA1c) among participants with T2D. Additionally, we conduct secondary analyses to explore whether the duration of having T2D may differentially affect beliefs depending on optimal versus non-optimal HbA1c levels. In addressing these goals, we aim to identify the belief-domain(s) that may pose barriers to necessary self-care behaviors with the vision of informing future interventions to promote better diabetes care and management.

## Methods

### Study design and population

This cross-sectional study was conducted online in Singapore between April–November 2021. To avoid response biases where participants’ ratings of their general life beliefs may bias or cloud their responses on health-related beliefs, the survey was administered over two parts. Part A included questions on general life beliefs and participant characteristics. Part B, administered a week later, focused more specifically on general health beliefs and condition-specific beliefs.

Given that many individuals with T2D commonly have other comorbidities (particularly, hypertension and/or hyperlipidemia; henceforth referred to as HT/HL)^31,33^, it is essential to ascertain whether the effects observed in this study are unique to T2D status or may be attributed to having a chronic condition in general. To investigate this possibility, we also recruited individuals with self-reported HT/HL—chronic diseases that are frequently present in both T2D and non-T2D populations—regardless of participants’ T2D status. This approach would allow us to examine whether mindsets and SE matter (a) only for individuals with T2D, (b) for those with T2D and HT/HL, and/or (c) those with just HT/HL. (Here, we wish to note that although self-reported conditions may be biased, individuals’ perception of whether they have a disease/condition may be a relevant determinant of psychological constructs, regardless of whether they have been clinically diagnosed.) Participants with severe or confounding conditions (cancers, chronic pain, heart attack/failure, kidney disease, liver failure, mental disorders, lupus, and stroke) were screened out at the start of the survey.

Furthermore, as age is a non-modifiable risk factor that may invariably influence the results of this study given the chronic conditions under investigation, we applied a minimum age of 40 to reduce self-selection bias based on age-associated health conditions. In determining an appropriate age cutoff, we first considered the typical onset age range of T2D, HT, and HL, and applied a minimum age that would reasonably serve as a “common denominator” across these chronic conditions. (There are no current standard guidelines on HT onset age although some sources define early-onset HT as occurring at ≤ 55 years of age^34^ (aligning with British HT guidelines recommending different first-line treatment for patients over and under 55 years of age^35^); other studies segment participants into different 10-year ranges of onset categories primarily based on convenience^36,37^. HL, on the other hand, is more common in individuals above the age of 40^38,39^). Second, we considered data collection constraints given that online research panels often skew towards younger participants. Setting a higher minimum age (e.g., cutoff age of 55) would severely limit our already constrained participant pool, especially after applying our health-related exclusion criteria. Taking these factors into consideration, a minimum age threshold of 40 for all participant groups would balance comparability across diseased and non-diseased groups and data collection feasibility concerns.

As this study was conducted during the Covid-19 pandemic, it was not feasible to gain access to patients with chronic diseases through local clinics and hospitals. We engaged a market research company who recruited potential participants via their research panel. Invitations to the study were also disseminated via newspaper ads and social media platforms to increase participation, especially from individuals with T2D. The study and its procedures were approved by the Institutional Review Board at the National University of Singapore (IRB reference: NUS-IRB-2020-101). All procedures were performed in accordance with the approved guidelines and regulations. Prospective participants were provided with the study information prior to the survey and informed about the use of their answers for analysis under anonymity. All participants provided their online informed consent before commencing the survey. Bot detection was implemented in the survey to facilitate identification and exclusion of potential non-human survey completions.

### Measures

#### General life self-efficacy beliefs

General life SE was assessed using the widely used New General Self-Efficacy scale (NGSE)^40^. Measuring beliefs in one’s overall ability or confidence to perform effectively across various general situations or tasks (e.g., “I believe I can succeed at most any endeavor to which I set my mind”), this 8-item scale has been previously demonstrated to have high construct validity and reliability^29^ and possessed high internal reliability in this study (α = 0.92). Responses were scored on a 5-point Likert scale (5 = strongly agree), with higher scores indicating greater general life SE.

#### Health self-efficacy

SE towards one’s general health was measured using the 8-item Perceived Health Competence Scale (PHCS; e.g., “I handle myself well with respect to my health”)^41^. The PHCS has been previously demonstrated to have high construct validity and reliability^30^ and possessed high internal reliability in this study (α = 0.81). Responses were scored on a 5-point Likert scale (5 = strongly agree), with higher scores indicating greater health self-efficacy.

#### Condition-specific self-efficacy

SE toward blood glucose, blood pressure, and cholesterol levels were each assessed using the 8-item Perceived Diabetes Self-Management Scale (PDSMS)^42^. Adapted from the PHCS, the PDSMS is a valid measure of diabetes SE that holds high validity and consistency, and has been recommended for use with other chronic conditions by replacing the word “diabetes” with the specific condition under investigation (e.g., “I handle myself well with respect to my *blood glucose*”)^42^. The PDSMS possessed good internal reliability in this study (α_high blood pressure_ = 0.85; α_high cholesterol_ = 0.73; α_blood glucose_ = 0.89). Responses were scored using the same 5-point Likert scale, with higher scores indicating greater SE toward the indicated condition.

#### General life mindset beliefs

General life GM was assessed using the domain-general measure of implicit theories^12^. This well-established 3-item scale measures whether one’s personal attributes are believed to be fixed or malleable (e.g., “The kind of person someone is is something very basic about them and it can’t be changed very much”) and possessed high internal reliability in this study (α = 0.86). Responses were scored on a 6-point scale (6 = very strongly disagree), with higher scores indicating a stronger general life GM.

#### Health mindset

Mindset towards one’s general health was measured using an implicit theories of intelligence scale that has been well validated with test–retest reliability^11,43^. Originally comprising statements pertaining to intelligence, this 4-item scale has been adapted to different domains by replacing “intelligence” with relevant keywords appropriate to the research context (e.g., weight^7^). Here, we used “health level” to reflect the degree to which one believes their health is changeable (e.g., “My health level is something about me that I can’t change very much”). This scale possessed high internal reliability in this study (α = 0.91). Responses were scored on a 6-point scale (6 = strongly disagree), with higher scores indicating a stronger GM regarding their general health.

#### Condition-specific mindset

Mindsets toward blood glucose, blood pressure, and cholesterol levels were measured using the same scale assessing general health mindsets. As before, “intelligence” was replaced with condition-specific words to reflect the degree to which one believes that their blood glucose, blood pressure, or cholesterol is changeable (e.g., “No matter how hard I try, I can’t really change my *glucose level*”). Each respective condition-specific mindset scale possessed high internal reliability (α_high blood pressure_ = 0.94; α_high cholesterol_ = 0.96; α_blood glucose_ = 0.96). Responses were scored on the same 6-point scale, with higher scores indicating a stronger GM toward that condition.

#### Other variables

As part of the survey, we assessed participant characteristics including age, sex, marital status, ethnicity, education, employment, religion, and housing type. Participants provided their height (cm) and weight (kg), from which we obtained their body mass index (BMI).

#### HbA1c and duration since T2D diagnosis

Since we were interested in exploring the relationship between beliefs and blood glucose levels, we asked participants with T2D to indicate their most recent HbA1c reading. Given the possibility that duration of T2D status may impact health beliefs, we also asked participants to indicate the year in which they were diagnosed with T2D.

### Statistical analyses

Statistical analyses and data visualization were performed using SPSS version 27 and R 4.1.0. Based on preliminary linear regression analyses, we suspected a violation of the normality assumption which was confirmed by Kolmogorov–Smirnov tests on the linear regression residuals (*p* < 0.001). As such, we used quantile regressions to examine whether and in which domains would GM and SE beliefs differ between participants with T2D and those without T2D.

We conducted quantile regression models for each belief-domain dependent variable (DV; e.g., GM towards general health). Each model initially comprised the following primary predictors: T2D status (0 = non-T2D; 1 = T2D), HT/HL status (0 = non-HT/HL; 1 = HT/HL), and their interaction (T2D × HT/HL). Including the interaction term allowed us to account for potential conditional effects (e.g., whether an effect of T2D on general health GM is dependent on HT/HL status). Subsequently, we dropped the interaction term if it was non-significant (*p*-value > 0.05) but included HT/HL status in the model to accurately isolate the effect of T2D while accounting for HT/HL. We controlled for age, sex, ethnicity, marital status, education, employment, housing type, religion, and BMI in all analyses.

To examine the relationships between beliefs (toward general health and blood glucose) and HbA1c among participants with T2D, we ran a nonparametric splines regression model for each belief–HbA1c relationship (natural cubic splines basis; three knots defined at the 15th, 50th, and 85th quantiles), adjusting for the same covariates as before. This model allowed us to identify the relationship structure and account for a possible non-linear structure.

It is plausible that individuals who have been diagnosed with T2D for longer may tend to have more negative mindsets and SE beliefs towards their health and/or blood glucose. To explore whether T2D duration has an effect on health and/or blood glucose beliefs and whether the effect might differ for those with ideal versus non-ideal glycemic control we conducted a quantile regression to assess the two-way effect of self-reported HbA1c and T2D duration on all four combinations of belief-domain scores. Subsequently, we dropped the interaction term from each quantile regression model if it was non-significant. As before, we controlled for the same covariates. For glycemic levels, we used a dichotomous indicator with HbA1c ≤ 6.4% as the threshold for ideal glycemic control in diabetes care management^1^. For T2D duration, we used a dichotomous indicator with a duration of ≤ 3 years as the cutoff for more recent diagnosis compared to those living with T2D for a longer time^44^.

## Results

### Participant characteristics

**Figure 1:**
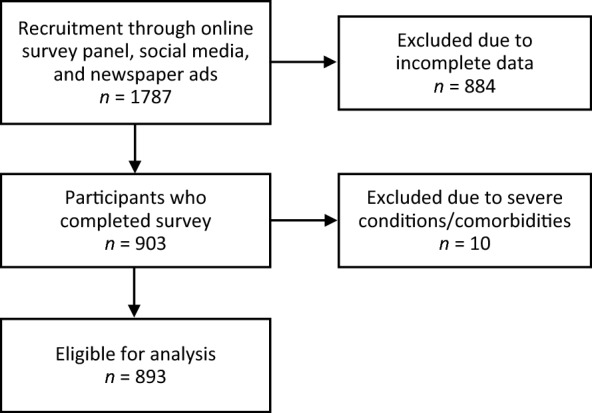
Flowchart of participant recruitment.

From the 1787 individuals who commenced the self-administered survey, a total of 903 participants completed the study (this sample attrition was due to the two-part study design as many dropped out before completing Part A or without participating in Part B). We excluded 10 participants who indicated other major/confounding conditions that were not automatically screened out at the start of the survey, leaving a total of 893 completed responses (see Fig. 1).

The final sample included 309 participants with T2D, of whom 189 had HT/HL comorbidities. Among the 584 participants without T2D, 314 of them had HT/HL and 270 participants were healthy with no pre-existing health conditions. Table 1 provides more details of our sample characteristics.

**Table 1:** Characteristics of participants (*n* = 893).

	T2D (*n* = 309)	Non-T2D (*n* = 584)	*p*-value	Total (*n* = 893)
	%/M ± SD	%/M ± SD		%/M ± SD
Sex				
Male	63.1%	58.0%	0.142	59.8%
Female	36.9%	42.0%		40.2%
Age (years)	53.8 ± 9.3	52.1 ± 9.1	0.007	52.7 ± 9.2
Ethnicity				
Chinese	79.9%	87.0%	0.004	84.5%
Malay	8.4%	4.6%		5.9%
Indian	9.7%	4.8%		6.5%
Other	1.9%	3.6%		3.1%
Education				
Secondary or less	12.5%	10.3%	0.212	11.1%
Pre-tertiary	23.6%	26.2%		25.3%
Other diploma	11.7%	11.6%		11.6%
University degree	36.2%	38.2%		37.6%
Postgraduate degree	15.9%	13.7%		14.4%
Marital status				
Unmarried*	26.5%	27.0%	0.099	26.9%
Married	73.5%	72.9%		73.1%
Employment				
Employed^†^	76.6%	86.1%	0.010	83.0%
Unemployed/Student	6.7%	3.1%		4.3%
Retired	13.6%	7.4%		9.5%
Homemaker	2.9%	3.4%		3.2%
Housing Type				
HDB^‡^ 1–3 room apt	15.2%	18.0%	0.032	17.0%
HDB 4 room apt	28.5%	32.2%		30.8%
HDB 5 room apt	29.4%	24.5%		26.2%
Private condo/apt	18.8%	21.4%		20.5%
Landed property	8.1%	3.9%		5.4%
Religion				
Buddhist/Taoist	34.3%	39.6%	0.104	37.7%
Christian/Catholic	34.7%	33.9%		34.2%
Muslim	8.7%	6.5%		7.3%
Hindu/Sikhism	6.1%	2.7%		3.9%
Other	16.2%	17.3%		16.9%
Body Mass Index (BMI)^	26.3 ± 5.2	24.1 ± 5.8	< 0.001	24.8 ± 5.7
Underweight: < 18.5	1.6%	5.7%		4.3%
Normal: 18.5–22.9	25.2%	38.9%		34.2%
Overweight: 23.0–29.9	54%	48.8%		50.6%
Obese: ≥ 30.0	19.1%	6.7%		11%
T2D duration	9.8 ± 9.2	–	–	–
3 years or less	28.8%	–	–	–
4–10 years	34.0%	–	–	–
More than 10 years	37.2%	–	–	–
High cholesterol and/or hypertension (*n* = 503)	61.2%	53.8%	0.034	56.3%
Most recent HbA1c % (*n* = 235)	7.2 ± 1.7	–	–	–
< 6.5%^δ^	31.5%	–	–	–
≥ 6.5%	68.5%	–	–	–

*Unmarried: single, widowed, divorced/separated.

^†^Employed: all occupational vocations including self-employment.

^‡^HDB: public residential housing under Singapore’s Housing & Development Board (HDB).

^BMI: BMI cut-off points for Asian populations.

^δ^HbA1c levels: values under 6.5% indicate ideal blood glucose levels^45^.

### Differences in mindset and self-efficacy beliefs between individuals with T2D and those without T2D

The T2D × HT/HL interaction term was not significant for all our belief-domain DVs (*p*s ≥ 0.395). We therefore excluded this interaction term in all quantile regression analyses reported in this study.

As seen in Table 2 (T2D column), there were no significant differences in general life GM and SE beliefs between individuals with T2D and individuals without T2D (*p*s ≥ 0.425). Instead, significant differences were observed in several health-related belief-domains. Compared to those without T2D, individuals with T2D were observed to have a significantly lower GM towards their blood glucose (*B*_adj_ = − 0.218, *p* = 0.041). Participants with T2D also had a significantly lower SE towards their general health (*B*_adj_ = − 0.118, *p* = 0.004), blood glucose (*B*_adj_ = − 0.131, *p* = 0.010), and cholesterol (*B*_adj_ = − 0.108, *p* = 0.024). There were no significant differences in GM towards general health, blood pressure, and cholesterol between individuals with T2D and those without T2D (*p*s ≥ 0.530). No significant differences were observed in SE beliefs toward blood pressure (*p* = 0.408). (See Supplementary Table S1 for correlational values between GM and SE beliefs across each domain.)

**Table 2:** Multivariate adjusted association of T2D status and HT/HL status with beliefs.

Beliefs (DVs)	T2D			HT/HL		
	Adjusted^a^ B	SE	p	Adjusted^a^ B	SE	p
Growth mindset						
General life	< 0.001	0.037	1.000	< 0.001	0.037	1.000
General health	− 0.031	0.081	0.700	0.144	0.079	0.068
Blood glucose	− 0.218	0.107	0.041	− 0.001	0.105	0.994
Blood pressure	0.064	0.102	0.530	0.041	0.100	0.682
Cholesterol	0.010	0.107	0.928	− 0.198	0.105	0.059
Self-efficacy						
General life	0.026	0.032	0.425	− 0.015	0.032	0.636
General health	− 0.118	0.041	0.004	− 0.023	0.040	0.561
Blood glucose	− 0.131	0.051	0.010	− 0.074	0.050	0.135
Blood pressure	− 0.041	0.050	0.408	0.000	0.049	0.992
Cholesterol	− 0.108	0.048	0.024	− 0.019	0.047	0.690

Note: Interactions of T2D × HT/HL were found to be non-significant and therefore excluded in these adjusted quantile regression analyses. The T2D column contains coefficients of the main effects comparing between T2D and non-T2D statuses; the HT/HL column contains coefficients of the main effects comparing between HT/HL and non-HT/HL statuses.

*B*, unstandardized slope coefficient; *SE*, standard error.

^a^Adjusted for age, BMI, gender, ethnicity, marital status, education, housing type, occupation, and religion.

No significant differences were observed between individuals with HT/HL and those without HT/HL across all the belief-domain constructs (see HT/HL column in Table 2).

### Relationship between beliefs and HbA1c

**Figure 2:**
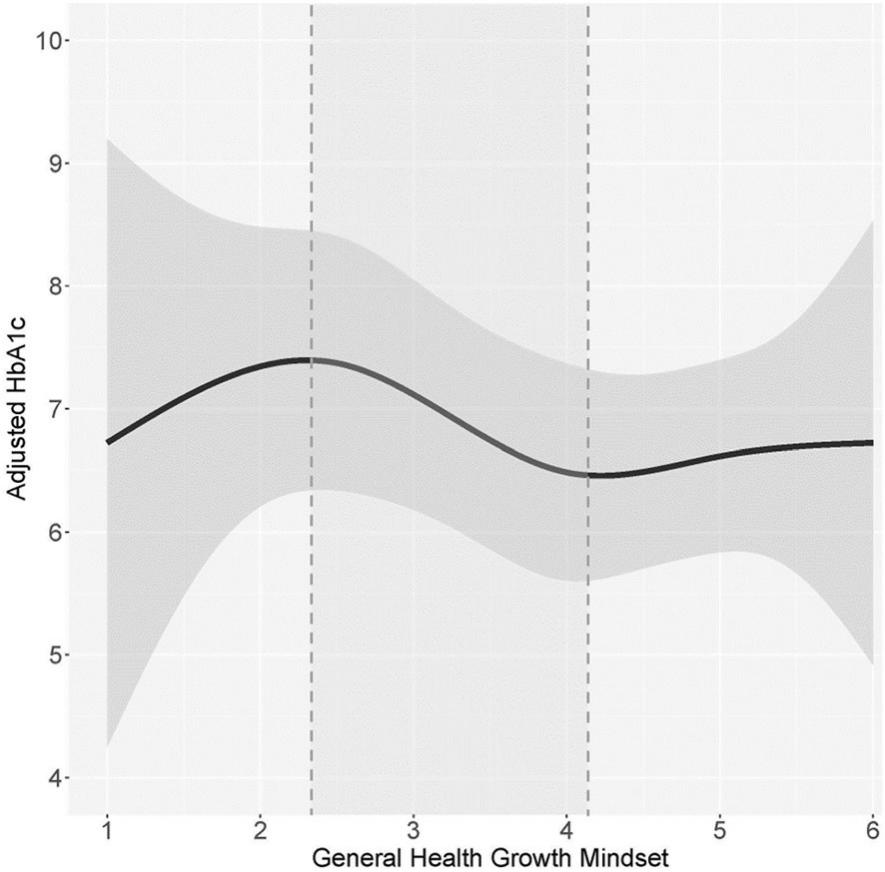
The association between general health growth mindset and HbA1c. The black line represents the nonparametric splines regression curve; the grey shaded area represents its corresponding 95% confidence band. Among participants with a general health GM score of 2.35–4.10, a higher GM is associated with lower HbA1c. Approximately 43.8% of participants with T2D who reported their HbA1c fall within this GM range. Among participants above the 4.10 GM threshold, there is no association between general health GM and HbA1c. We omit the interpretation of the pattern of association for GM scores under 2.35 due to the very wide confidence bands caused by limited data points in those lower ranges.

**Figure 3:**
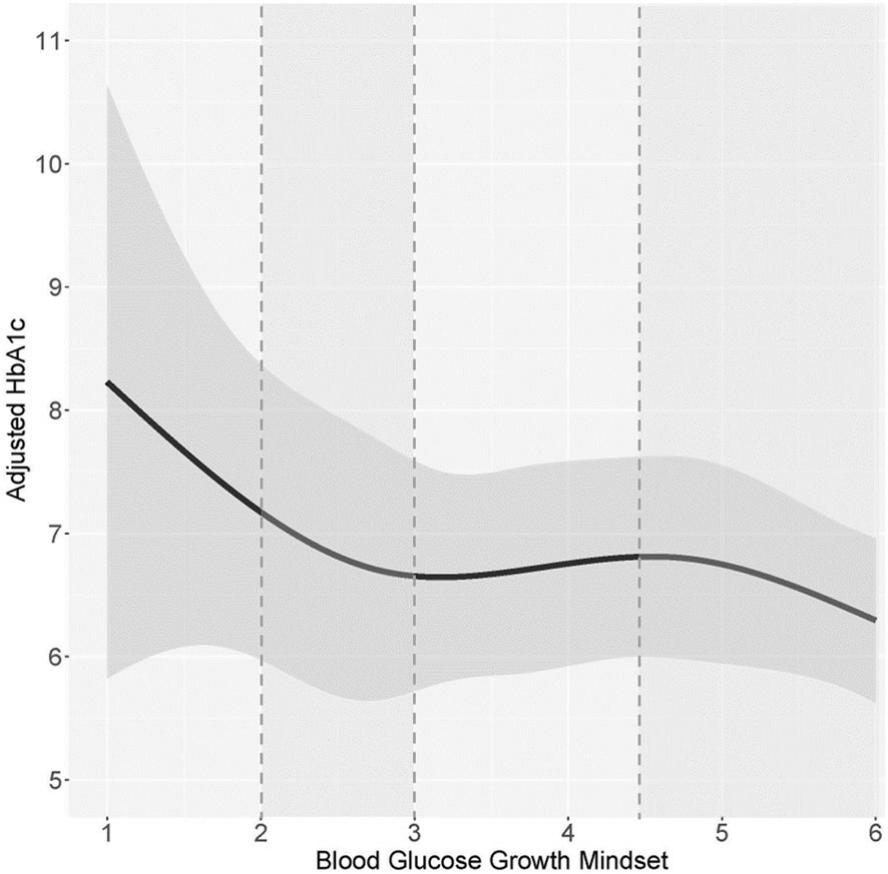
The association between blood glucose GM and HbA1c. We exercise caution in the interpretation of the pattern of association for GM scores under 2.00 due to the very wide confidence bands caused by limited data points in those lower ranges. Among participants with a GM score between 2.00 and 3.00, the results show that higher blood glucose GM is associated with lower HbA1c. Approximately 20.4% of participants with T2D who reported their HbA1c fall within this GM range. Participants with moderate blood glucose GM (3.01–4.50) have minimal variation in their HbA1c. Among those with high GM (≥ 4.51), higher blood glucose GM is associated with lower HbA1c. Approximately 45.5% of participants who reported their HbA1c fall within this range.

**Figure 4:**
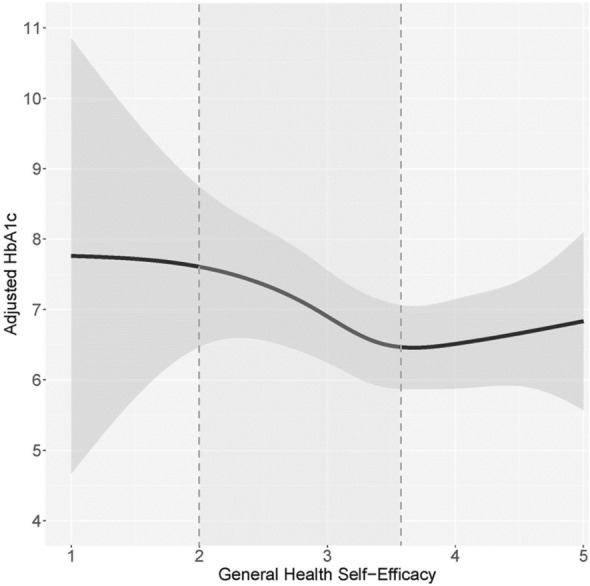
The association between general health SE and HbA1c. We exercise caution in the interpretation of the pattern of association for SE scores under 2.00 due to the very wide confidence bands caused by limited data points in those lower ranges. Among participants with a general health SE score between 2.00 and 3.51, higher SE is associated with lower HbA1c. Approximately 62.6% of participants who reported their HbA1c fall within this SE range. There is no association between general health SE and HbA1c among participants above the 3.51 SE threshold.

**Figure 5:**
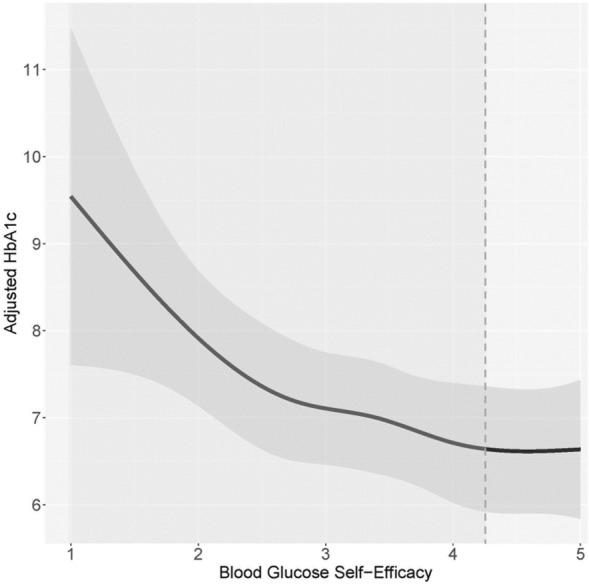
The association between blood glucose SE and HbA1c. Higher blood glucose SE is associated with lower HbA1c among participants with a SE score below 4.30. Approximately 90.2% of participants who reported their HbA1c fall within this SE range. There is no association between blood glucose SE and HbA1c among participants scoring above the 4.30 SE threshold.

Based on the nonparametric splines regression curves for GM and SE beliefs toward general health and blood glucose, we identified thresholds ranges for which the patterns of association differ substantially (see shaded regions in Figs. 2, 3, 4 and 5).

### T2D duration

The dichotomous T2D duration x HbA1c interaction term was not significant for all four belief-domain DVs (*p*s ≥ 0.078). Controlling for HbA1c without the interaction term, individuals with longer T2D duration (i.e., more than 3 years) had a lower median GM towards their blood glucose compared to those with shorter T2D duration (*B*_adj_ = − 0.471, *p* = 0.021). T2D duration had no significant effect on general health GM, general health SE, and blood glucose SE (*p* ≥ 0.283). (Sensitivity analysis by adjusting the T2D duration cutoff to 5 years or more is presented in the Supplementary material. The results show that changing the cutoff to 5 years or more does not change any of our conclusions.)

## Discussion

In this study, we found associations between T2D status and specific health-related GM and SE beliefs. Specifically, our results revealed that the median GM towards the malleability of one’s blood glucose was significantly lower in individuals with T2D than those without T2D. We particularly note that differences in GM were observed only in the domain specific to diabetes—i.e., blood glucose—without extending to beliefs toward general life, general health, blood pressure, and cholesterol levels. In terms of SE, the results indicated that compared to individuals without T2D, those with T2D had lower SE toward their general health, blood glucose, and cholesterol. No SE differences were observed between T2D and non-T2D individuals pertaining to general life and blood pressure domains. These non-significant SE results suggest that (a) general life encompasses a broad range of non-health related situations that extend beyond the immediate concerns of T2D management and (b) managing blood pressure may be perceived as less complex and demanding compared to blood glucose control (whereby a sense of diminished SE is felt in light of recurring setbacks and suboptimal outcomes), explaining why SE toward blood pressure did not differ between those with T2D and those without T2D.

While one might expect to observe consistent significant associations on the same domains across both GM and SE constructs (e.g., if there are significant associations between T2D status and *SE towards cholesterol*, we should also observe significant associations between T2D status and *GM towards cholesterol*), the differences between GM and SE beliefs within specific health domains demonstrate nuances that distinguish these psychological constructs. For instance, beliefs that one’s cholesterol levels can be improved (GM) does not mean that one feels equally confident and equipped in performing the necessary actions for that change or improvement to happen (SE).

Furthermore, there were no associations between HT/HL status and any of the belief-domain constructs. One plausible explanation for these findings is that managing HT/HL is often less complex and demanding in terms of lifestyle adaptations compared to T2D, underscoring why individuals with HT/HL perceive the belief-domains similarly to those without HT/HL. The observed differences in health beliefs, therefore, point towards T2D in particular, rather than the presence of HT/HL, in relation to having lower SE and GM towards specific aspects of their health.

Notably, given that blood glucose was the only domain consistently affected by T2D status across both GM and SE beliefs,individuals with T2D may struggle particularly with managing their blood glucose compared to managing their blood pressure or cholesterol levels. While high blood pressure and cholesterol may be more easily managed with medication adherence (with efficacy that is often sustained over time), medications to reduce blood glucose are less successful without lifestyle modifications^32,46^ (e.g., when one’s diet continues to be high in simple carbohydrates and sugars). Moreover, blood glucose levels tend to worsen with increased duration of T2D, requiring ever more intensive pharmacological treatment and lifestyle modification. By implication, the challenge of managing blood glucose seems to rest substantially on the complexity of self-management behaviors of continued effort and readjustments, not just as an individual in functional daily living, but also within the interpersonal and social environment^2,46^. By extension, our findings may be generalizable to other chronic diseases (e.g., heart failure, chronic obstructive airway disease, gouty arthritis) in which patients face recurring setbacks or that require self-care behaviors beyond medication adherence.

Although the effect sizes of T2D on the scores for the significant GM and SE health-related domains were not large, the scores were not homogenous across all individuals with T2D. Among participants with T2D, we observed a pattern of elevated HbA1c among those with lower general health and blood glucose GM or SE scores. Higher GM and SE scores were associated with lower HbA1c of a nadir of 6.3–6.7%. Namely:

- A low general health GM score of 2.30 had a corresponding HbA1c of 7.4%, while a higher GM score of 4.10 had a corresponding HbA1c of 6.5%.
- A low blood glucose GM score of 2.00 had a corresponding HbA1c of 7.2%, while the highest GM score of 6 had a corresponding HbA1c of 6.3%.
- A low general health SE score of 2.00 had a corresponding HbA1c of 7.7%, while a higher SE score of 3.50 had a corresponding HbA1c of 6.5%.
- A low blood glucose SE score of 1.00 had a corresponding HbA1c of 9.5%, while a higher SE score of 4.2 had a corresponding HbA1c of 6.7%.

These observations suggest that patients with lower levels of GM and SE beliefs may benefit from improvements in these respective belief-domains (i.e., general health GM and SE; blood glucose GM and SE). Naturally, our findings prompt next questions regarding causality: is high HbA1c the result of negative health-related beliefs, or do negative health-related beliefs arise from high HbA1c levels? While it is conceivable that a reciprocal relationship exists, where HbA1c levels and health-related beliefs influence and reinforce each other (e.g., patients with elevated HbA1c might develop a greater FM toward managing their health; this FM, in turn, may hinder their engagement in necessary self-care behaviors, manifesting in a negative cycle of suboptimal HbA1c), we acknowledge that our study’s correlational design precludes claims about causality and prompts the need for prospective investigations to further ascertain the dynamics of these relationships.

The exploratory results on T2D duration offer further nuance on individuals with T2D who might be more susceptible to fatalistic beliefs towards their blood glucose and suggest that lower GM beliefs may be “acquired” over time. Namely, those who have had T2D for more than 3 years tend to have less of a GM towards their blood glucose, regardless of their HbA1c control. These individuals comprise a sizeable segment who could potentially benefit from appropriate interventions on the malleability of their condition.

Because beliefs influence motivation and behavior in ways that can profoundly affect health outcomes^47,48^, it may be crucial to target beliefs that hinder self-management by equipping individuals with T2D to develop a greater GM and SE towards their blood glucose (e.g., beliefs in the potential to change, that specific conditions are malleable, that improvements stem from effort and learning from “mistakes,” that failures are not indicative of one’s identity or potential). Perhaps one way to break the vicious “negative beliefs ↔ poor glucose control” cycle is to address their mindsets toward diabetes and self-management efficacy. Doing so could potentially reduce psychological barriers toward necessary lifestyle modifications and medication adherence and thereby lead to improvements in HbA1c, reinforcing further self-efficacy and growth mindset beliefs in a positive cycle of change and diabetes control. Another approach is to employ short-term interventions that lead to visible decreases in blood glucose levels (e.g., supervised short-term dietary changes alongside continuous glucose monitoring or treatment intensification among patients at more advanced stages of the disease) to provide the needed physiological and psychological breakthroughs for individuals who believe that their blood glucose levels cannot be easily managed, disrupting their FM about the non-malleability of diabetes control.

### Future research directions

Our findings emphasize several important areas for future efforts to explore how GM can be fostered among those with T2D. First, a crucial next step would be to explore and establish any causal relationship(s) between GM and T2D outcomes using experimental studies (e.g., targeted mindset interventions to evaluate shifts in health and behavioral outcomes relative to a control group). Second, we need an improved understanding of the areas in which individuals with T2D feel resistant to change and action (i.e., specific health-related or self-management areas where individuals tend to hold a FM). Third, complementary research should identify key aspects or features of GM that would most effectively address those FM areas (e.g., should emphasis be placed on the malleability of their *condition*, on affirming *effort* over outcomes, on how *mistakes* should be reframed, on how *setbacks* can serve as stepping-stones, on how *feedback* informs improvements, or their combination?). Fourth, although intervention effectiveness relies predominantly on patient participation, healthcare providers play a major role in influencing patients’ perspectives and intentions. For instance, making subtle changes in physician–patient conversations by framing information differently (e.g., emphasizing specific strengths of the patient that may increase confidence in making lifestyle adjustments or nudging patients toward a GM by saying, “Your blood glucose level is not just dependent on your genes; it can improve when you make small changes to your diet.”) can provide the psychological opportunities conducive for GM and SE beliefs to thrive^48,49^.

### Limitations

This present study is not without limitations. First, given the cross-sectional design of the survey, we do not claim causality of the effects observed nor conclude that increasing GM and/or SE would certainly lead to blood glucose improvements. Instead, our data show patterns of relationships within certain thresholds whereby participants with a certain level of GM or SE were observed to have a corresponding HbA1c percentage. Future experimental studies using GM/SE interventions and randomized controlled trials would help to address this limitation and provide evidence on important causal relationships between beliefs and health/behavioral outcomes including HbA1c, improved dietary choices, and increased physical activity. Longitudinal studies may also be valuable to ascertain the effectiveness of GM/SE interventions over the duration of patients’ T2D journey to better tailor the content, frequency, and implementation mode of such interventions.

Second, there is a possibility of self-selection bias due to the online nature of data collection during the Covid-19 pandemic. Our sample, therefore, may comprise different ethnic proportions and more educated participants compared to the diabetic population in Singapore. For instance, while the majority of participants with T2D in our study are of Chinese ethnicity, figures from a 2010 National Health Survey in Singapore reported that Indians had the highest prevalence of diabetes among the ethnic groups, followed by Malays and Chinese^50^. Data from a number of participants were also excluded due to incomplete responses to the two-part study. We assume that there were no systematic differences between participants who were excluded due to incomplete responses and participants who were included in the final analysis. However, there may be an inherent general difference in mindsets and self-efficacy between participants who completed the survey and those who dropped out—a limitation that we are unable to account for at present.

Third, our analyses were based on self-reported health conditions, belief-domain ratings, and HbA1c levels (the latter only among participants with T2D who were able to recall their most recent HbA1c results). Hence, we are unable to validate the actual conditions, beliefs, and HbA1c levels that they claim to have. We also do not have data on the dates of their latest HbA1c results and acknowledge that many non-critical healthcare visits would have been delayed or forgone during the Covid-19 pandemic. Hence, the reliability of these self-reported values should be approached with caution. Fourth, the study was limited to participants residing in Singapore, a country with a 14.9% diabetes prevalence in adults aged 20–79 and with one of the highest proportions of total deaths associated with diabetes (29%)^1^. It would be valuable to replicate similar studies in multiple countries, particularly in regions where T2D has become of urgent concern to confirm and extend the generalizability of the findings.

Fifth, we also acknowledge that there are other psychological concepts that may be related to the concept of GM and SE—such as health LOC and outcome expectancies—that were not included in this paper. This may potentially limit our understanding of whether and to what extent these concepts intersect or interact with GM and SE, and how they might impact downstream health-related behaviors and outcomes.

Taken together, this study breaks new ground in exploring whether and where GM and SE beliefs matter for people living with T2D, a condition that has become a growing global emergency^51^. Importantly, knowing that such beliefs—consistently and particularly towards blood glucose—tend to be significantly lower amongst individuals with T2D provides important findings in explaining why managing diabetes may be particularly difficult for those with T2D. Evidence from this study offers a premise for future studies to not only probe the causality relationship between negative beliefs and blood glucose control, but also informs the direction and development of evidence-based behavioral change interventions.

### Supplementary Information

Supplementary Information.

## Data Availability

The dataset generated and analyzed during the current study is not publicly available due to the Data Management Policy under the approved IRB but is available from the corresponding author on reasonable request.
