# Supplementary material for: Mindsets and self-efficacy beliefs among individuals with type 2 diabetes
Source: Sci Rep. 2023 Nov 21;13:20383. doi: 10.1038/s41598-023-47617-4 (PMC10663547; doi:10.1038/s41598-023-47617-4)
Supplement: Supplementary file 1 — Supplementary Information. [file 41598_2023_47617_MOESM1_ESM.pdf]

Mindsets and self-efficacy beliefs among individuals with type 2 diabetes

Carolyn J. Lo*
carolyn.lo@nus.edu.sg
Yong Loo Lin School of Medicine, National University of Singapore, Singapore.
LRF Institute for the Public Understanding of Risk, National University of Singapore, Singapore.

Leonard Lee
leonard.lee@nus.edu.sg
LRF Institute for the Public Understanding of Risk, National University of Singapore, Singapore.
Department of Marketing, NUS Business School, National University of Singapore, Singapore.

Weichang Yu
weichang.yu@unimelb.edu.au
School of Mathematics and Statistics, University of Melbourne, Australia.

E Shyong Tai
mdctes@nus.edu.sg
Department of Medicine, National University Hospital, Singapore.
Yong Loo Lin School of Medicine, National University of Singapore, Singapore.

Tong Wei Yew
mdcyewtw@nus.edu.sg
Department of Medicine, National University Hospital, Singapore.
Yong Loo Lin School of Medicine, National University of Singapore, Singapore.

Isabel L. Ding
isabel.ding@wbs.ac.uk
Warwick Business School, University of Warwick, UK.

*Corresponding author

Supplementary Information for

Mindsets and self-efficacy beliefs among individuals with type 2 diabetes

Carolyn Lo, Leonard Lee, Weichang Yu, E Shyong Tai, Tong Wei Yew, Isabel Ding

Corresponding author: Carolyn Lo
Email: carolyn.lo@nus.edu.sg

This supplementary file includes:

1. Supplementary Table S1
2. Supplementary note: T2D duration (cutoff under 5 years)

Supplementary Table S1. Adjusted correlation between growth mindset and self-efficacy beliefs in general life, general health, and condition-specific domains

**Domain**	***Adjusted[1] correlation***	***p***
General life	0.025	0.475
General health	0.508	< 0.001
Blood glucose	0.568	< 0.001
Blood pressure	0.463	< 0.001
Cholesterol	0.573	< 0.001

[1]Adjusted for age, BMI, gender, ethnicity, marital status, education, housing type, occupation, and religion.

Supplementary note

T2D duration (cutoff under 5 years). The dichotomous T2D duration (cutoff: < 5 years) x HbA1c (cutoff: ≤ 6.4%) interaction term was not significant for all the four belief-domain DVs (*p*s ≥ 0.109). Controlling for HbA1c without the interaction term, individuals with longer T2D duration (5 years or more) had a lower median GM towards their blood glucose compared to patients with shorter T2D duration (i.e., had T2D for less than 5 years; $B_{adj} = -0.631$, $p < 0.001$). T2D duration had no significant effect on general health GM, general health SE, and blood glucose SE ($p \geq 0.080$).
